# Population Structure and Antimicrobial Resistance of Invasive Serotype IV Group B *Streptococcus*, Toronto, Ontario, Canada

**DOI:** 10.3201/eid2014.140759

**Published:** 2015-04

**Authors:** Sarah Teatero, Allison McGeer, Aimin Li, Janice Gomes, Christine Seah, Walter Demczuk, Irene Martin, Jessica Wasserscheid, Ken Dewar, Roberto G. Melano, Nahuel Fittipaldi

**Affiliations:** Public Health Ontario, Toronto, Ontario, Canada (S. Teatero, A. Li, J. Gomes, C. Seah, R.G. Melano, N. Fittipaldi);; University of Toronto, Toronto (A. McGeer, R.G. Melano, N. Fittipaldi);; Mount Sinai Hospital, Toronto (A. McGeer, R.G. Melano);; Public Health Agency of Canada, Winnipeg, Manitoba, Canada (W. Demczuk, I. Martin);; McGill University, Montreal, Quebec, Canada (J. Wasserscheid, K. Dewar);; Genome Quebec Innovation Centre, Montreal (J. Wasserscheid, K. Dewar)

**Keywords:** bacterial infection, invasive bacterial disease, group B *Streptococcus*, streptococci, Streptococcus agalactiae, bacteria, serotype IV, multilocus sequence typing, whole-genome sequencing, antimicrobial resistance, population structure, Toronto, Canada

## Abstract

We recently showed that 37/600 (6.2%) invasive infections with group B *Streptococcus* (GBS) in Toronto, Ontario, Canada, were caused by serotype IV strains. We report a relatively high level of genetic diversity in 37 invasive strains of this emerging GBS serotype. Multilocus sequence typing identified 6 sequence types (STs) that belonged to 3 clonal complexes. Most isolates were ST-459 (19/37, 51%) and ST-452 (11/37, 30%), but we also identified ST-291, ST-3, ST-196, and a novel ST-682. We detected further diversity by performing whole-genome single-nucleotide polymorphism analysis and found evidence of recombination events contributing to variation in some serotype IV GBS strains. We also evaluated antimicrobial drug resistance and found that ST-459 strains were resistant to clindamycin and erythromycin, whereas strains of other STs were, for the most part, susceptible to these antimicrobial drugs.

## Abstract 2

Conjugate vaccines should include polysaccharide or virulence proteins of this serotype to provide complete protection.

Group B *Streptococcus* (GBS), also known as *Streptococcus agalactiae*, are a major cause of neonatal sepsis and meningitis and are increasingly being associated with invasive infections in nonpregnant adults (*1*–*3*). For instance, in 2011, adult cases accounted for nearly 90% of the burden of GBS disease in the United States (*4*). Elderly persons and those with preexisting conditions (e.g., diabetes mellitus, cirrhosis, cancer, and compromised immunity) are most at risk (*5*). The clinical features of GBS disease in adults range from localized tissue infection to severe bacteremia with shock (*1*). Less common clinical syndromes, such as endocarditis and meningitis, are associated with considerable illness and death (*1*).

GBS are classified into 10 serotypes (Ia, Ib, and II–IX) on the basis of a serologic reaction against capsular polysaccharide. The most common GBS serotypes causing invasive disease in the United States and Canada in adults and neonates are Ia, III, and V (*2*,*3*,*6*–*9*). However, recent reports have shown that serotype IV GBS is emerging in pregnant carriers and causing infections in neonates and adults in North America and several other regions (*10*–*14*). This emergence is of concern because GBS conjugate vaccines that are being developed to prevent invasive disease may protect only against serotypes Ia, Ib, II, III, and V, or combinations thereof (*15*,*16*).

Ferrieri et al. reported different genetic backgrounds among serotype IV GBS strains, which have been shown to include sequence types (STs) ST-452 and ST-459 (*11*). Horizontal gene transfer occurs frequently in GBS. For example, capsular switching from serotype III to IV has been recently reported and found to be the result of recombination involving the entire *cps* locus (*8*,*17*,*18*). GBS are considered to be universally susceptible to penicillins; therefore, these are the primary drugs used for prophylaxis and treatment of GBS disease (*19*,*20*). However, macrolide and lincosamide antimicrobial drugs, such as erythromycin and clindamycin, are often used to treat GBS infections in patients allergic or suspected to be allergic to β-lactam drugs. Acquisition of resistance against these drugs by horizontal gene transfer has been frequently described in GBS (*2*,*21*).

We recently reported a relatively high prevalence (6.2%) of serotype IV strains among GBS causing invasive infections in Toronto, Ontario, Canada (*8*). In the current study, we used whole-genome sequencing (WGS) to characterize the population structure of serotype IV GBS isolates. We describe a genetically heterogeneous population of isolates that differ in their antimicrobial drug resistance profiles.

## Methods

### Bacterial Strains, Culture Conditions, DNA Preparations, Serotyping, and Pilus Typing

We used 37 serotype IV GBS strains collected during 2009–2012 by the Toronto Invasive Bacterial Disease Network, a population-based surveillance system for invasive bacterial diseases in metropolitan Toronto and the Peel region in Ontario, Canada (total population under surveillance ≈5.5 million persons in 2011). Laboratory-based surveillance involves the 28 hospitals providing care to and the 25 laboratories processing sterile site cultures for residents of the population area; laboratory personnel submit all GBS isolates from sterile sites to the central study laboratory. The sample was composed of all available serotype IV GBS strains determined by latex agglutination and corresponded to 6.2% of GBS identified during the study by the program (*8*). Only 1 strain per patient was included in the collection; there was no clear evidence of localized outbreaks caused by serotype IV GBS during the collection period. Strains are listed in Technical Appendix Table 1.

Bacteria were grown at 37°C in an atmosphere of 5% CO_2_ on Columbia agar plates supplemented with 5% sheep blood. Genomic DNA was obtained by using the QIAamp DNA Minikit (QIAGEN, Toronto, Ontario, Canada) according to the manufacturer’s protocol for gram-positive organisms. The presence of pilus islands PI-1, PI-2a, and PI-2b was determined by using PCR as described (*22*).

### WGS, Bioinformatics Methods, and Phylogenetic Analysis

WGS libraries were prepared for all 37 isolates by using Nextera XT Kits (Illumina, San Diego, CA, USA) and sequenced as paired-end reads with either Illumina HiSeq 2500 (100 bp + 100 bp) or MiSeq (150 bp + 150 bp) instruments (data are available at the Sequence Read Archive under accession no. SRP040805). Parsing of the multiplexed sequencing reads and removal of barcode information was performed by using onboard software. STs were derived directly from short reads by using SRST2 (https://github.com/katholt/srst2). Ambiguous or novel alleles were confirmed by PCR and Sanger sequencing.

Neighbor-joining phylogenetic trees (1,000 bootstrap replications) were then generated by using SplitsTree4 (*23*) and the concatenated sequences of the 7 loci used in the GBS multilocus sequence typing (MLST) scheme. For WGS-based phylogenies, we sequenced to closure the genomes of ST-459 strain NGBS061 and ST-452 strain NGBS572 (GenBank accession nos. CP007631 and CP007632, respectively) by using single-molecule real-time (SMRT) sequencing (Pacific Biosciences, Menlo Park, CA, USA). Two SMRT cells of sequence were generated for each isolate, which generated >220 Mb of data in reads >3 kb (37,218 reads, average length 6.1 kb for NGBS061; 54,803 reads, average length 5.6 kb for NGBS572). In brief, we used HGAP version 2 (*24*) to correct the long reads and Celera Assembler 7.0 (*25*) to assemble the corrected reads, followed by 2 rounds of polishing with Quiver (https://github.com/PacificBiosciences/GenomicConsensus). Coverage of final assemblies in reads >3 kb was 84× for NGBS061 and 111× for NGBS572.

To assess base-calling accuracy in the Pacific Biosciences assembly, Illumina short reads for the 2 isolates were aligned to their respective assemblies by using BLAT (*26*). Both genome assemblies were completely concordant with full-length perfectly aligning Illumina short-reads, except for NGBS061 in a region of increased AT richness, which had reduced Illumina coverage. For consistency, both genome assemblies were formatted to begin at the first nucleotide of the intergenic region immediately preceding the gene encoding the chromosomal replication initiation protein (*dnaA*; Genbank accession no. YP-328728).

Illumina short reads of all other isolates were aligned to the reference genomes, and coverage relative to the reference genome was calculated by using Mosaik (https://code.google.com/p/mosaik-aligner/). Polymorphisms were identified by using VAAL (*27*). A matrix file containing genotypes of all strains at each polymorphic locus was created from the VAAL polymorphism output data by using a custom script. Then, for each individual strain, single-nucleotide polymorphisms (SNPs) were concatenated in order of occurrence relative to the genome of the corresponding reference strain (see Results). Neighbor-joining phylogenetic trees (1,000 bootstrap replications) were then generated with SplitsTree4.

The A5 pipeline was used for de novo assembly of Illumina-sequenced GBS strains (*28*). Areas of recombination of the ST-196 strains NGBS447 and NGBS472 were defined by using BratNextGen software (*29*) run with 20 iterations and 100 replicates, using a p value of 0.05 as the significance cutoff. Genome visualizations were created by using BRIG (*30*) and edited by using Adobe Illustrator (Adobe Systems Inc., San Jose, CA, USA).

### Antimicrobial Drug Susceptibility

MICs for penicillin, ampicillin, cefotaxime, levofloxacin, tetracycline, erythromycin, and clindamycin were determined by using the agar dilution method according to the Clinical and Laboratory Standards Institute (*31*). We also used SRST2 and a database of 1,913 variants of genes encoding antimicrobial drug resistance (https://github.com/katholt/srst2) to test for genetic determinants of antimicrobial drug resistance in genomes of serotype IV strains.

## Results

### Isolate Source and Age Distribution of Patients

The 37 serotype IV GBS isolates used in this study represent 6.2% of 600 GBS strains isolated in the Toronto–Peel metropolitan area of Ontario during 2009–2012 (*8*). One isolate was collected in 2009, 15 isolates in 2010, 9 isolates in 2011, and 12 isolates in 2012. Most isolates were from invasive cases in adults (19–59 years of age, n = 14) or older adults (>60 years of age, n = 21) patients. We did not observe serotype IV GBS in neonates with early-onset disease (<7 days of age). However, 1 isolate was obtained from a child with late-onset disease (7–89 days of age), and an additional strain was isolated from a child 188 days of age. Most (34/37) serotype IV isolates were collected from blood; only 1 isolate was collected from soft tissue, and 2 isolates were collected from synovial fluid.

### Genetic Diversity of Serotype IV GBS Strains

**Figure 1:**
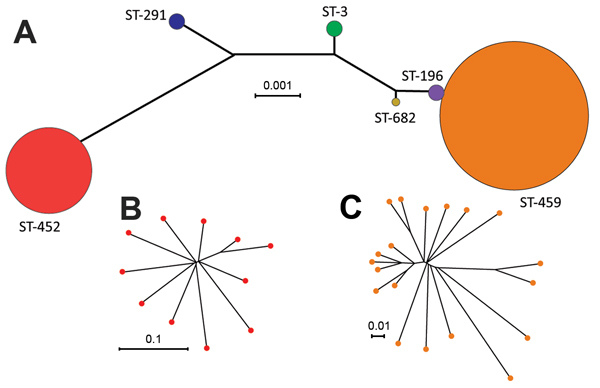
Inferred genetic relationships of invasive serotype IV group B *Streptococcus* (GBS), Toronto, Ontario, Canada. A) Neighbor-joining phylogenetic tree constructed by using the concatenated sequences of the 7 gene loci (*sdhA*, *adhP*, *tkt*, *glcK*, *atr*, *pheS*, and *glnA*) used in the multilocus sequence typing (MLST) scheme for GBS. Each circle represents a single MLST sequence type (ST); circle colors differentiate the 6 STs found among strains in our collection and their sizes are proportional to the number of isolates. B) Neighbor-joining phylogenetic analysis based on concatenated sequences of 316 nonredundant single-nucleotide polymorphism (SNP) loci identified by whole-genome sequencing relative to the core genome ST-452 reference strain NGBS572, which shows further diversity within ST-452. C) Diversity within ST-459 serotype IV GBS shown by neighbor-joining phylogenetic analysis based on concatenated sequences of 526 nonredundant SNP loci relative to the core genome of ST-459 reference strain NGBS061. Scale bars indicate nucleotide substitutions per site.

To assess genetic diversity of serotype IV GBS strains in our collection, we sequenced the genomes of all 37 strains and used SRST2 to derive MLST information directly from the short-read WGS data. We identified 6 STs representing 3 clonal complexes (CCs), which is indicative of a relatively high level of genetic diversity among our isolates. Two isolates were ST-291 included in hypervirulent CC17; 11 isolates were ST-452 (CC23); 2 isolates were ST-3 (CC1); and 1 isolate was a novel ST-682 included in CC1. Most (19/37) strains belonged to ST-459 (CC1). We also identified 2 strains of ST-196, a closely related single locus variant (*sdhA* gene) of ST-459. The 2 STs comprising most strains (ST-452 and ST-459) were also the most genetically distant, as shown by neighbor-joining phylogenetic analysis of concatenated sequences of alleles at all MLST loci (Figure 1, panel A).

To further understand genetic differences between these 2 STs, we arbitrarily chose 1 ST-452 strain (NGBS572) and 1 ST-459 strain (NGBS061) and sequenced their genomes to closure by using Pacific Biosciences SMRT technology. The genomes were circular chromosomes of 2,061,426 bp and 2,221,207 bp, respectively (Technical Appendix Figure 1). We did not find evidence of any associated plasmids in either isolate. However, we used an SMRT sequencing protocol with a DNA size-selection step for DNA fragments >20 kb, which might have removed small plasmids that could have been present. The difference in genome size between the strains was explained primarily by the smaller number and size of mobile genetic elements found in NGBS572 than in NGBS061 (Technical Appendix Figure 1). The core genomes of NGBS572 and NGBS061 (i.e., ≈1.9-Mbp portion of the genome lacking mobile genetic elements that is conserved in gene content between both strains) differed by 13,158 biallelic SNPs and 852 insertions or deletions (indels).

We next investigated genetic diversity within the ST-452 and ST-459 groups. Both groups were relatively homogeneous. On average, ST-452 strains differed from the ST-452 reference strain NGBS572 by only 45 SNPs and 10 indels in the core genome. On average, ST-459 strains had only 68 SNPs and 16 indels relative to the core genome of the ST-459 reference strain NGBS061. Neighbor-joining phylogenetic trees constructed by using concatenated SNPs in the core genome showed additional but limited diversity among isolates within each ST and some clustering within ST-459 (Figure 1, panels B, C).

Other STs in our collection had either 1 or 2 strains. Thus, we chose not to sequence these genomes to closure to assess genetic diversity but instead identified polymorphisms relative to the ST-452 and ST-459 reference strains. Both ST-3 strains showed a comparable number of polymorphisms against each reference strain (Technical Appendix Table 2). Similar results were observed for each of the ST-291 strains (i.e., both strains differed from the ST-452 and ST-459 reference strains by a comparable number of polymorphisms) (Technical Appendix Table 2). In contrast, the 2 ST-196 strains greatly differed between themselves in the number of polymorphisms separating them from the reference strains (Technical Appendix Table 2). One ST-196 strain, strain NGBS447, had 384 core SNPs and 60 core indels relative to the ST-459 reference strain, where the second ST-196 strain, NGBS472, had 1689 core SNPs and 124 core indels relative to the ST-459 reference strain. Further analysis discovered that the excess polymorphisms in NGBS472 were not evenly distributed across the genome. Instead, some discrete regions of the genome showed an overabundance of polymorphisms, which is suggestive of recombination.

Using BratNextGen, we precisely defined these regions of recombination (Technical Appendix Figure 2). When these regions of recombination were excluded from the analysis, strain NGBS447 had 107 SNPs and 16 indels relative to the ST-459 reference strain and NGBS472 had 281 SNPs and 128 indels relative to the same reference genome. Genome regions having undergone recombination in strain NGBS472 included genes encoding major virulence factors, such as the 2-component system *csrRS* (*32*) and pili (*33*). Pili in GBS are encoded by genes found in 2 distinct genome locations and organized in so-called pilus island 1 (PI-1) and PI-2. PI-2 has 2 variants (PI-2a or PI-2b) (*33*). One of the ST-196 strains had a pilus profile of PI-1 + PI-2a, and the other had a profile of PI-1 + PI-2b (Technical Appendix Figure 3). Except for these ST-196 strains, strains of the different STs had consistent pilus island profiles (Technical Appendix Figure 3).

### Antimicrobial Drug Susceptibility of Serotype IV GBS Strains

**Figure 2:**
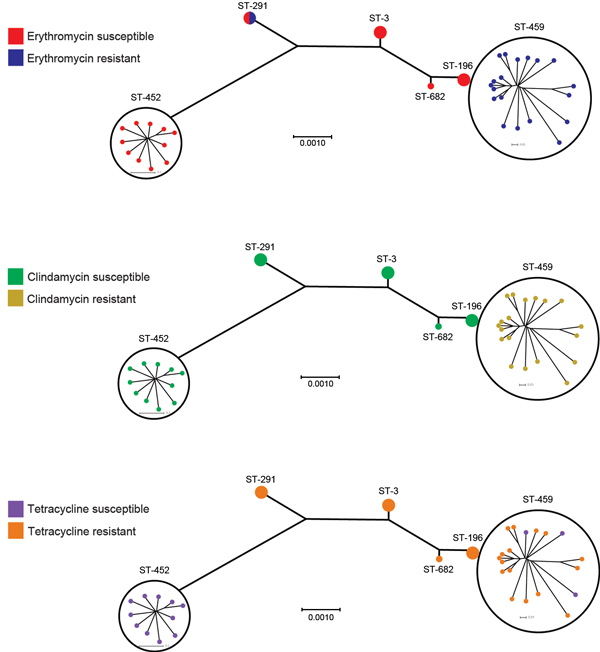
Susceptibility to erythromycin, clindamycin, and tetracycline among serotype IV group B *Streptococcus* and sequence types (STs), Toronto, Ontario, Canada. All ST-459 strains and 1 ST-291 strain were resistant to erythromycin; we detected *ermTR* and *ermT* genes in all erythromycin-resistant strains (Technical Appendix). Resistance to clindamycin was observed only among ST-459 strains. Resistance to tetracycline was common among all STs except ST-452 and correlated with presence of the *tetM* gene in isolates (Technical Appendix). Scale bars indicate nucleotide substitutions per site.

All serotype IV GBS strains were susceptible to all β-lactams, vancomycin, and levofloxacin. We observed resistance to erythromycin and clindamycin in 20/37 (54%) and 19/37 (51%) of the isolates, respectively; 23 (62%) of the isolates were resistant to tetracycline. The proportion of resistance and susceptibility, as well as 50% MICs and 90% MICs, are shown in Technical Appendix Table 3. All ST-459 isolates were resistant to clindamycin and erythromycin; ST-452 isolates were susceptible to these antimicrobial drugs (Figure 2; Technical Appendix Table 4). Strains of other STs were susceptible to these 2 drugs, except 1 of the ST-291 strains, which was resistant to erythromycin but susceptible to clindamycin (Figure 2; Technical Appendix Table 4). Macrolide resistance in ST-459 strains is probably attributable to the action of *ermTR*, a gene that was detected in all ST-459 strains (Technical Appendix Table 1). We also identified the *ermT* gene in the genome of the erythromycin-resistant ST-291 strain (Technical Appendix Table 1). Resistance to tetracycline correlated with presence of the *tetM* gene (Technical Appendix Table 1) and was observed among strains of all STs except ST-452. However, we identified 3 ST-459 strains that were susceptible to tetracycline (Figure 2; Technical Appendix Table 4). One of these had the *tetM* gene, but genome data showed a 7-nt deletion in the gene, which was predicted to result in early termination of translation. We did not detect *tet* genes in the other 2 ST-459 strains (Technical Appendix Table 1).

## Discussion

The prevalence of serotype IV GBS is increasing globally among pregnant women; infections in neonates and invasive infections in adults are also increasing. Large proportions of serotype IV GBS isolates have been reported among carrier isolates in the United Arab Emirates (26.3%) and Turkey (8.3%) (*34*,*35*). In Zimbabwe, serotype IV strains accounted for 5.1% of GBS isolates from carrier pregnant women and 4.0% of all GBS strains isolated from hospitalized patients (*36*). A recent report found a relatively high percentage (16%) of serotype IV strains among cases of GBS early-onset disease in Minnesota (*11*). In a longitudinal study in the United States, the proportion of this serotype among all GBS strains isolated from nonpregnant adults increased from 0.2% in 1998–1999 to 5.7% in 2005–2006 (*3*).

In Canada, a country-wide survey conducted in 1996 found no serotype IV GBS cases in 79 nonpregnant adult women (*9*). However, we recently reported that serotype IV GBS in the greater Toronto area during 2009–2012 accounted for 6.2% of all invasive GBS infections (*8*). Cases in children were rare, and most (94%) serotype IV GBS invasive infections in Toronto were in adults >19 years of age. It may be possible that the low incidence of serotype IV in neonates, and the absence of cases of early-onset disease, is a reflection of a low prevalence of colonizing serotype IV GBS in women of childbearing age. Future studies to determine the prevalence of serotype IV GBS in pregnant women will be useful in elucidating the reasons for this apparent absence of serotype IV GBS neonatal invasive disease.

Given the restricted geographic and temporal origin of our collection of invasive serotype IV GBS strains, we hypothesized that their emergence might represent the successful expansion of a single clone. However, other GBS serotypes usually encompass strains with different genetic backgrounds that might have different tissue tropism and virulence potential (*37*). Thus, an alternative hypothesis was that our serotype IV GBS collection was composed of strains of diverse genetic backgrounds and that >1 clones underwent expansion.

To begin to differentiate between these and other hypotheses, we conducted WGS and evaluated the population structure of our collection. Results showed a relatively high level of genetic diversity among the 37 serotype IV GBS isolates, which were assigned to 6 MLST STs and 3 CCs. Two of our invasive serotype IV isolates belonged to ST-291, a member of the hypervirulent CC17. Previous studies have shown that several strains of this ST have acquired a serotype IV capsule by recombination involving exchange of the full capsule-encoding *cps* locus (*8*,*17*). We also found 2 ST-3 strains and a novel ST-682 strain, both included in CC1, one of the major CCs associated with human GBS disease. Our collection contained 2 strains of ST-196 (CC1). WGS showed that the genomes of these 2 ST-196 strains differed extensively, and further analysis indicated that these genome differences were likely caused by recombination. These findings highlight the major role of horizontal gene exchange of extensive regions of DNA in GBS diversification (*38*) and expose the limitations of MLST to fully identify GBS diversity.

ST-452 (CC23) and ST-459 (CC1) were the 2 most common STs in our collection. These 2 STs have recently been described and were found in invasive serotype IV strains that caused disease in Minnesota (*10*,*11*). The reasons why these 2 highly divergent genomic backgrounds seemingly thrive among serotype IV GBS strains that caused invasive disease in Toronto remain to be investigated. However, SNP analysis showed a low level of genetic diversity among strains of each ST-452 and ST-459, which is compatible with recent emergence and ongoing clonal expansion. These STs were also the most phylogenetically distant groups in our collection. We showed that ST-452 and ST-459 genomes differ greatly in size, largely because they have different mobile genetic elements, such as prophages and different pilus islands. The core genomes of these 2 most prevalent STs also differed considerably, as shown by SNP analysis.

Another difference among the groups was that ST-459 strains were resistant to clindamycin and erythromycin, and for the most part to tetracycline, whereas ST-452 isolates were susceptible to these drugs. We associated resistance to macrolides with presence of the *ermTR* gene in all ST-459 strains. The increase in the number of cases of invasive disease caused by serotype IV isolates and concurrent emergence of clindamycin resistance among a successful clone of this serotype could be an early indication of the beginning of clinical problems similar to those observed for serotype V GBS (*39*). Resistance to erythromycin was observed in 1 ST-291 strain and correlated with presence of the *ermT* gene. Tetracycline resistance was observed in 23 (62%) of the serotype IV GBS isolates, and in most instances correlated with presence of the *tetM* gene.

Capsular switching has been described frequently in GBS (*22*,*40*). At least in part, our results suggest that different lineages of serotype IV GBS have independently acquired a serotype IV capsule from unidentified donors by recombination. As the global presence of serotype IV continues to increase, monitoring of this emerging serotype is warranted. Conjugate vaccines that are being developed should include polysaccharide and/or associated GBS virulence proteins of this serotype, or they risk not being completely protective against the full range of GBS disease.

**Technical Appendix.** Additional information regarding population structure and antimicrobial drug susceptibility of invasive serotype IV group B *Streptococcus*, Toronto, Ontario, Canada.
