# Correction: Vol. 21, No. 4

**DOI:** 10.3201/eid2108.C12108

**Published:** 2015-08

**Keywords:** errata, erratum, correction

An incorrect version of the Technical Appendix was provided online for the article Population Structure and Antimicrobial Resistance of Invasive Serotype IV Group B *Streptococcus*, Toronto, Ontario, Canada (S. Teatero et al.). The article has been corrected online (http://wwwnc.cdc.gov/eid/article/21/4/14-0759_article).

