# Supplementary material for: Population Structure and Antimicrobial Resistance of Invasive Serotype IV Group B Streptococcus, Toronto, Ontario, Canada
Source: Emerg Infect Dis. 2015 Apr;21(4):585–91. doi: 10.3201/eid2014.140759 (PMC4378482; doi:10.3201/eid2014.140759)
Supplement: Supplementary file 1 — Technical Appendix. Additional information regarding population structure and antimicrobial drug susceptibility of invasive serotype IV group B Streptococcus, Toronto, Ontario, Canada. [file 14-0759-Techapp-s1.pdf]

Article DOI: http:/dx.doi.org/10.3201/eid2104.140759

Population Structure and Antimicrobial Drug Susceptibility of Invasive Serotype IV Group B *Streptococcus*, Toronto, Ontario, Canada

Technical Appendix

Technical Appendix Table 1. Serotype IV group B *Streptococcus* strains included in this study, Toronto, Ontario, Canada*

Strain	Year isolated	ST	Source	*ermA* allele†	*ermC* allele†	*ermT* allele†	*tetM* allele†
NGBS024	2009	459	Blood	*ermA* 2	–	–	*tetM* 13
NGBS046	2010	459	Synovial fluid	*ermA* 2	–	–	*tetM* 13
NGBS049	2010	459	Blood	*ermA* 2	–	–	*tetM* 13
NGBS058	2010	459	Blood	*ermA* 2	–	–	*tetM* 13‡
NGBS061	2010	459	Blood	*ermA* 2	–	–	*tetM* 2
NGBS065	2010	452	Blood	–	–	–	-
NGBS070	2010	459	Blood	*ermA* 2	–	–	*tetM* 2
NGBS100	2010	452	Blood	–	–	–	–
NGBS122	2010	452	Blood	–	–	–	–
NGBS146	2010	459	Blood	*ermA* 2	–	–	*tetM* 13
NGBS151	2010	3	Blood	–	–	–	*tetM* 13
NGBS187	2010	452	Blood	–	–	–	–
NGBS191	2010	459	Blood	*ermA* 2	–	–	*tetM* 13
NGBS197	2010	452	Blood	–	–	–	–
NGBS199	2010	459	Blood	*ermA* 2	–	–	–
NGBS258	2011	459	Blood	*ermA* 2	–	–	*tetM* 13
NGBS290	2010	459	Blood	*ermA* 2	–	–	–
NGBS314	2011	452	Blood	–	–	–	–
NGBS367	2011	291	Blood	–	*ermC* 12	*ermT* 2	*tetM* 7
NGBS379	2011	3	Blood	–	–	–	*tetM* 2
NGBS400	2011	459	Blood	*ermA* 2	–	–	*tetM* 2
NGBS410	2011	459	Blood	*ermA* 2	–	–	*tetM* 2
NGBS447	2011	196	Blood	–	–	–	*tetM* 13
NGBS472	2011	196	Blood	–	–	–	*tetM* 7
NGBS493	2012	459	Blood	*ermA* 2	–	–	*tetM* 13
NGBS507	2012	459	Blood	*ermA* 2	–	–	*tetM* 13
NGBS521	2012	459	Blood	*ermA* 2	–	–	*tetM* 13
NGBS525	2011	459	Soft tissue	*ermA* 2	–	–	*tetM* 13
NGBS528	2012	459	Blood	*ermA* 2	–	–	*tetM* 13
NGBS556	2012	452	Blood	–	–	–	-
NGBS562	2012	291	Blood	–	–	–	*tetM* 7
NGBS572	2012	452	Synovial fluid	–	–	–	–
NGBS588	2012	682	Blood	–	–	–	*tetM* 13
NGBS597	2012	452	Blood	–	–	–	–
NGBS598	2012	452	Blood	–	–	–	–
NGBS612	2012	452	Blood	–	–	–	–
NGBS615	2012	459	Blood	*ermA* 2	–	–	*tetM* 13

*ST, Sequence type; –, gene not detected by SRTST2.
†Allele number was determined on the basis of antimicrobial resistance database provided with SRST2 (https://github.com/katholt/srst2).
‡The *tetM* gene of this strain has a 7-bp deletion at position 640, which is predicted to result in early termination of translation.

Technical Appendix Table 2. Number of polymorphisms relative to serotype IV group B *Streptococcus* ST-452 and ST-459 reference strains, Toronto, Ontario, Canada*

		Reference strain			
		NGBS572 (ST-452)		NGBS061 (ST-459)	
Strain	ST	SNPs	Indels	SNPs	Indels
NGBS367	291	7,986	717	12,325	928
NGBS562	291	6,886	319	11,768	608
NGBS151	3	11,474	351	1,088	97
NGBS379	3	11,756	420	1,116	92
NGBS588	682	9,709	656	7,411	948
NGBS447	196	12,922	1,057	384	60
NGBS472	196	13,814	872	1,689	124

*Identified using VAAL (*1*). ST, sequence type; SNPs, single-nucleotide polymorphisms; Indels, insertions/deletions.

Technical Appendix Table 3. Susceptibility profiles of serotype IV group B *Streptococcus* isolates, Toronto, Ontario, Canada*

	No. isolates (%)			MIC (µg/mL)		
Drug	**Resistant**	**Intermediate**	**Susceptible**	**MIC_{50}**	**MIC_{90}**	**Range**
Tetracycline	23 (62)	0	14 (38)	64	64	<0.12–64
Ampicillin	0	0	37 (100)	0.25	0.25	0.12–0.25
Clindamycin	19 (51)	0	18 (49)	>8	>8	0.12–>8
Erythromycin	20 (54)	0	17 (46)	>8	>8	0.12–>8
Cefotaxime	0	0	37 (100)	0.06	0.06	0.03–0.06
Penicillin	0	0	37 (100)	0.06	0.06	0.03–0.06
Vancomycin	0	0	37 (100)	0.5	0.5	0.25–0.5
Levofloxacin	0	0	37 (100)	≤0.5	≤0.5	≤0.5–1

*MIC_{50}, 50% MIC; MIC_{90}, 90% MIC.

Technical Appendix Table 4. Antimicrobial drug resistance among group B *Streptococcus* sequence types, Toronto, Ontario, Canada*

	Tetracycline		Clindamycin		Erythromycin	
Sequence type	Susceptible	Resistant	Susceptible	Resistant	Susceptible	Resistant
ST-3	0	2	2	0	2	0
ST-196	0	2	2	0	2	0
ST-291	0	2	2	0	1	1
ST-452	11	0	11	0	11	0
ST-459	3	16	0	19	0	19
ST-682	0	1	1	0	1	0

*ST, sequence type.

Reference

1. Nusbaum C, Ohsumi TK, Gomez J, Aquadro J, Victor TC, Warren RM, et al. Sensitive, specific polymorphism discovery in bacteria using massively parallel sequencing. Nat Methods. 2009;6:67–9. PubMed http://dx.doi.org/10.1038/nmeth.1286

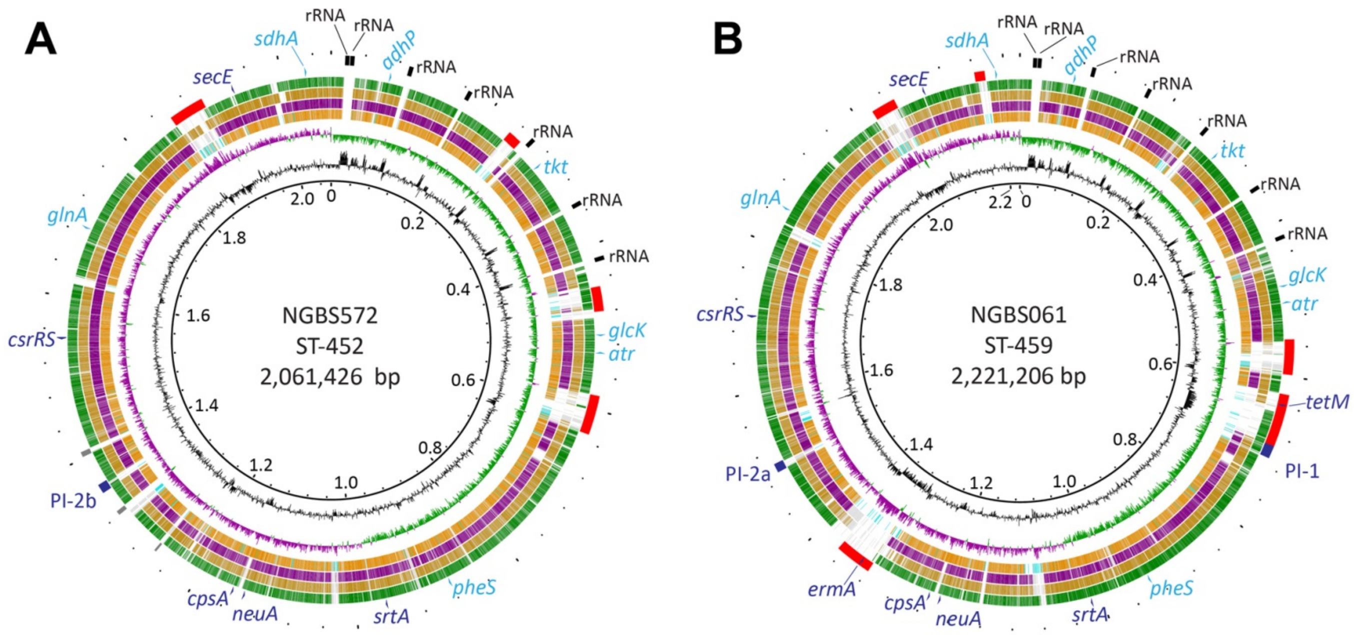

Technical Appendix Figure 1. Genome atlases of serotype IV group B *Streptococcus* strains, Toronto, Ontario, Canada. A) Strain NGBS572 (sequence type [ST]–452). Data from the innermost to outermost circles are in the following order: genome size in MP (circle 1); % G + C content (circle 2); GC skew or (G – C)/(G + C) averaged over a moving window of 10,000 bp, with excess G and excess C shown in green and purple, respectively (circle 3); TBLASTN comparison of the genome of the strain with the genome of ST-459 strain NGBS061 (circle 4, shades of gold represent the degree of homology); TBLASTN comparison of the genome of the strain with the genome of ST-25 strain NEM316 (GenBank accession no. NC_004368.1) (circle 5, shades of purple represent the degree of homology); TBLASTN comparison of the genome of the strain with the genome of ST-110 strain 2603V/R (GenBank accession no. NC_004116.1) (circle 6, shades of brown represent the degree of homology); TBLASTN comparison of the genome of the strain with the genome of ST-7 strain A909 (GenBank accession no. NC_007432.1) (circle 7, the shades of green represent the degree of homology). The outermost circle depicts in blue genome landmarks such as pilus islands, *srtA*, *secE*, *csrRS*, first and last genes of the *cps* locus (*cpsA* and *neuA*), and other genes of interest. Genes used for multilocus sequence typing are indicated in light blue. Putative mobile genetic elements are represented in red. B) Strain NGBS061 (ST-459). Data are the same as described for panel A, except that circle 4 is the TBLASTN comparison of the genome of the strain with the genome of ST-452 strain NGBS562.

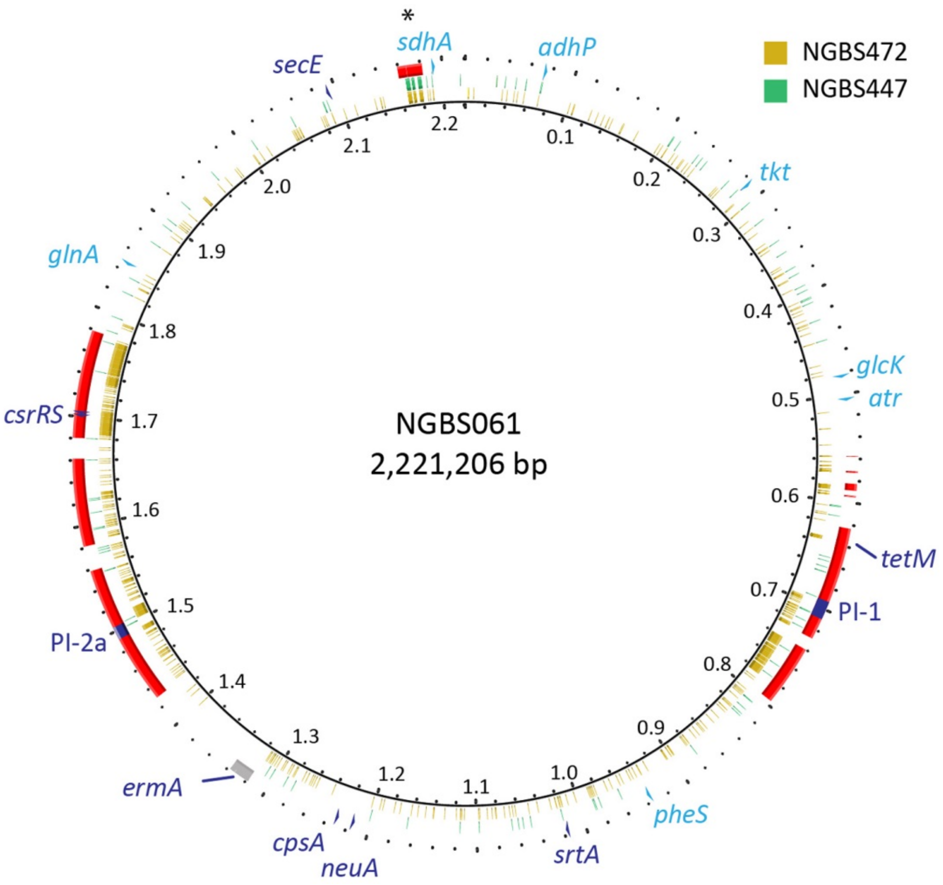

Technical Appendix Figure 2. BratNextGen Bayesian analysis of whole-genome data suggests recombination in sequence type (ST)–196 serotype IV Group B *Streptococcus* strains, Toronto, Ontario, Canada. Polymorphisms for ST-196 strains NGBS447 (green) and NGBS472 (gold) are plotted against ST-459 reference strain NGBS061. A total of 384 single-nucleotide polymorphisms (SNPs) and 60 indels were observed across the genome of strain NGBS447. For the most part, these polymorphisms were evenly distributed across the genome. However, a 24-kb region (indicated by the asterisk), encoding phage-related proteins, had an overabundance of polymorphisms suggestive of recombination, which was confirmed by using BratNextGen Bayesian analysis. Strain NGBS472 had also undergone recombination in this area. In addition, more polymorphisms (1,689 SNPs and 124 indels) were identified in this later strain relative to the ST-459 genome. Uneven distribution of these polymorphisms was observed, with most polymorphisms found concentrated in large, discrete areas of the NGBS472 genome. BratNextGen Bayesian analysis identified these areas of the NGBS472 genome, which are indicated in red in the outermost circle, as having undergone recombination. These regions encode, among others, genes potentially involved in virulence, such as polysaccharide biosynthesis machinery, ABC transporters, surface-anchored proteins, pili, and the global virulence 2-component regulatory system *csrRS*. When regions of recombination are excluded, NGBS447 had 107 SNPs and 16 indels and NGBS472 had 281 SNPs and 128 indels compared with the ST-459 reference genome. The outermost circle in blue indicates genome landmarks such as pilus islands, *srtA*, *secE*, *csrRS*, first and last genes of the *cps* locus (*cpsA* and *neuA*), and other genes of interest. Genes used for multilocus sequencing typing are indicated in light blue. Putative mobile genetic elements are indicated in gray.

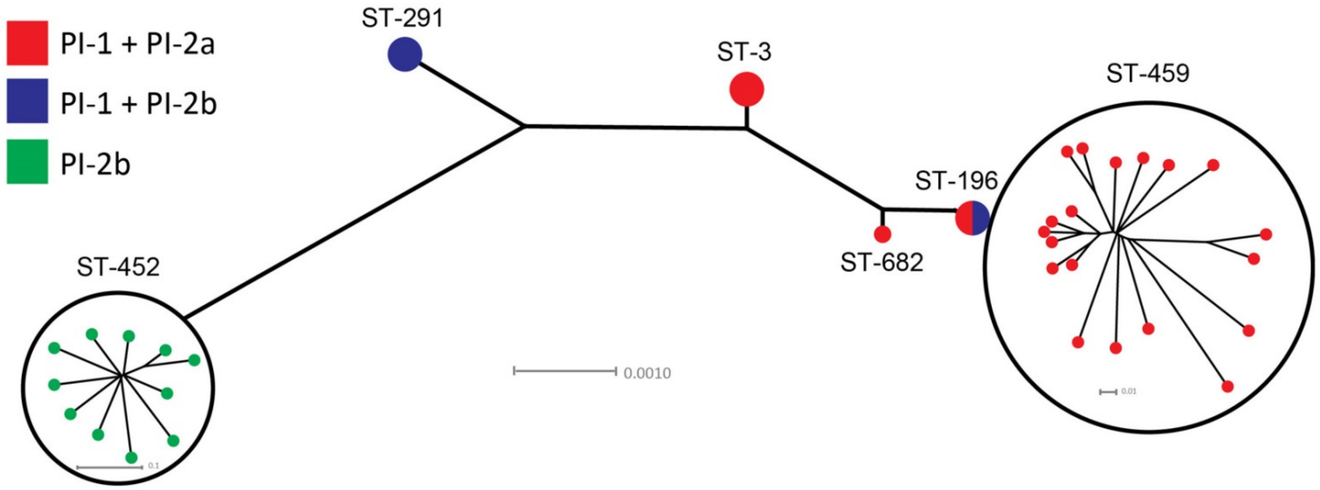

Technical Appendix Figure 3. Pilus island (PI) profiles among the different serotype IV Group B *Streptococcus* strains and sequence types (STs), Toronto, Ontario, Canada. A close association was observed between pilus profile and particular STs. All ST-452 strains had PI-2b but lacked pilus PI-1. ST-291 strains had PI-1 + PI-2b, which is the typical profile among clonal complex 17 strains. All strains belonging to ST-3, ST-682, and ST-459 had a profile PI-1 + PI-2a. However, the 2 strains in ST-196 had different pilus profiles. One ST-196 strain had the profile PI-1 + PI-2a and the other had PI-1 + PI-2b. Recombination events affecting vast parts of the genome of this second ST-196 strain were detected by using BratNextGen Bayesian analysis. See the text and online Technical Appendix Figure 2 for further details.
